# Metal-Diazo Radicals of α-Carbonyl Diazomethanes

**DOI:** 10.1038/srep22876

**Published:** 2016-03-10

**Authors:** Feifei Li, Longqiang Xiao, Lijian Liu

**Affiliations:** 1 Department of Polymer Science, College of Chemistry and Molecular Sciences, Wuhan University, Wuhan 430072, China

## Abstract

Metal-diazo radicals of α-carbonyl diazomethanes are new members of the radical family and are precursors to metal-carbene radicals. Herein, using electron paramagnetic resonance spectroscopy with spin-trapping, we detect diazo radicals of α-carbonyl diazomethanes, induced by [Rh^I^Cl(cod)]_2_, [Co^II^(por)] and PdCl_2_, at room temperature. The unique quintet signal of the Rh-diazo radical was observed in measurements of α-carbonyl diazomethane adducts of [Rh^I^Cl(cod)]_2_ in the presence of 5,5-dimethyl-pyrroline-1-N-oxide (DMPO). DFT calculations indicated that 97.2% of spin density is localized on the diazo moiety. Co- and Pd-diazo radicals are EPR silent but were captured by DMPO to form spin adducts of DMPO-N∙ (triplet-of-sextets signal). The spin-trapping also provides a powerful tool for detection of metal-carbene radicals, as evidenced by the DMPO-trapped carbene radicals (DMPO-C∙, sextet signal) and 2-methyl-2-nitrosopropane-carbene adducts (MNP-C∙, doublet-of-triplets signal). The transformation of α-carbonyl diazomethanes to metal-carbene radicals was confirmed to be a two-step process *via* metal-diazo radicals.

Radicals are highly reactive intermediates, long sought because of their importance in understanding reaction mechanisms and finding new reactions^1^. However, their extremely short lifetimes make their direct detection difficult and impede the development of radical chemistry. Carbenes, from nitrogen-releasing diazo compounds^2^, are the versatile active species in a broad range of reactions, including C-H functionalization^3^,^4^, carbon-carbon bond formation^5^,^6^, cyclopropanation^7^,^8^ and polycarbene construction^9^,^10^,^11^. Usually, these reactions are carried out with metal catalysts, and the intermediates, generally considered to be metal-carbenes, have attracted much attention over the past decade^12^,^13^,^14^,^15^,^16^. Recent studies have shown that some metal-carbenes within paramagnetic metal catalysts (such as Co^II^, Rh^II^, and Ir^II^) are carbon-centered metal-carbene radicals, not closed-shell metal-carbenes^17^,^18^,^19^. As such, their metal-diazo-adduct precursors are reasonably postulated to also possess radical character.

Because of the vigorous N_2_ evolution and high efficiencies of these reactions, few studies have reported such radicals^20^,^21^,^22^,^23^. The EPR signals of some diazo radical cations of diphenyldiazomethanes and their derivatives have been recorded after electrochemical oxidation or irradiation in Freon matrices^24^,^25^,^26^. In frozen toluene at 40 K, a carbon-centered Co-carbene radical was detected by EPR spectroscopy, along with several other paramagnetic cobalt species^17^, which complicated the spectral analysis. Diazo radicals may coexist with carbene radicals within reaction systems, and distinguishing them from one another is likewise difficult. Thus, the detection of metal-diazo radicals and their transformations into metal-carbene radicals remain unsolved challenges in diazo chemistry.

To overcome these challenges, we employed room-temperature electron paramagnetic resonance (RT-EPR) spectroscopy with a spin-trapping technique^27^, which offers a convenient and powerful way to identify both diazo radicals and carbene radicals and to simultaneously investigate their behaviors. Because of the persistent radical effects of the redox-active metal and nitroxide^1^, the transient radical intermediates can be either directly detected by EPR or captured by radical traps (Fig. 1). In this paper, we report that the use of [RhCl(cod)]_2_ (cod = 1,5-cyclooctadiene) allowed the RT-EPR detection of a rather stable dinitrogen radical of α-carbonyl diazomethane as a unique quintet signal. Density function theory (DFT) calculations support the EPR analyses, assigning a significant amount of spin density (97.2%) to the diazo moiety. To our knowledge, this work represents the first unequivocal spectroscopic confirmation of a metal-diazo radical. The diazo radicals generated by the [Co(por)] (por = *meso*-tetraphenylporphine)- and PdCl_2_-catalyzed systems were trapped by 5,5-dimethylpyrroline-1-*N*-oxide (DMPO) and the signals of their spin adducts (DMPO-N·) were detected by RT-EPR as typical triplet-of-sextets signals, which can be easily distinguished from the sextet EPR signal derived from the DMPO-trapped carbene radical adducts (DMPO-C·). Remarkably, with another strongly electrophilic spin trap (2-methyl-2-nitrosopropane, MNP), only the carbene radical with a single proton was trapped and detected as a highly distinctive doublet-of-triplets. By tracing the changes of these EPR signals over time, we confirmed that the metal-mediated transformation of α-carbonyl diazomethane into metal-carbene radical is a two-step process with a metal-diazo radical, which releases nitrogen gas and leaves its unpaired electron to the metal-carbene radical, as the key intermediate.

## Results

RT-EPR spectroscopy with spin-trapping was used to characterize the systems consisting of α-carbonyl diazomethane (methyl diazoacetate (MDA), ethyl diazoacetate (EDA), phenyl diazoacetate (PDA), and diazoacetophenone (DAP)) with [Rh^I^Cl(cod)]_2_, [Co^II^(por)] and PdCl_2_. All EPR samples were handled under a nitrogen atmosphere. Control experiments revealed that in the absence of metal catalysts, α-carbonyl diazomethanes and the spin traps, no EPR signals could be detected (Supplementary Fig. S1). In this way, the quintet EPR signals of the dinitrogen radical, the sextet signal of the DMPO-trapped carbene radical (DMPO-C·) and the doublet-of-triplets signal of the MNP-trapped carbene radical (MNP-C·) were recorded directly, whereas the triplet-of-sextets signal of the DMPO-trapped diazo radical (DMPO-N·) was distinguished from the background of DMPO-N· and DMPO-C· signals.

### Quintet EPR signal of dinitrogen radical

Remarkably, in all of the tested [RhCl(cod)]_2_-, [Co(por)]- and PdCl_2_-catalyzed systems in the presence of DMPO, only the [RhCl(cod)]_2_-catalyzed α-carbonyl diazomethane systems showed a striking quintet EPR spectrum (Fig. 2a), which obviously arises from coupling of two^14^ N-nuclei triplets^24^. Further simulation of the EPR spectrum obtained from the [RhCl(cod)]_2_-PDA system (Fig. 2b, detected at 1 min) revealed hyperfine coupling constants (HFC) of a^N^ = 15.00 and 17.00 G for the two different^14^ N-nuclei of the diazo group (Supplementary Fig. S2). However, the spectrum displays some lack of symmetry, partly due to the radical motion in solvent, and more importantly as a consequence of the superposition by other paramagnetic species in low concentration.

### Sextet EPR signal of DMPO-trapped carbene radical (DMPO-C·)

In the presence of DMPO, we detected a prominent sextet, as well as a three-line (1:1:1) aminoxyl radical signal (DMPOX) (Fig. 2c, [RhCl(cod)]_2_-PDA system, detected at 30 min). The sextet EPR signal showing HFCs of a^N^ = 14.16 G and a^H^ = 20.57 G (Supplementary Fig. S3), was assigned to the DMPO-trapped carbon-centered radical adduct (DMPO-C·)^28^. The appearance of the DMPOX signal with a single nitrogen HFC of a^N^ = 13.83 G was possibly due to the interaction of the metal-centered paramagnetic species with DMPO^29^. For reference, we noted that a scandium triflate system ([Sc(oTf)]-EDA) showed only the three-line DMPOX signal (Supplementary Fig. S4).

As shown in Fig. 2d, the quintet EPR signal was replaced by multiple EPR signals when the [RhCl(cod)]_2_-PDA system was measured after 5 min. Interestingly, the complicated resultant spectrum could be accurately simulated (red line in Fig. 2d) as a combination of the quintet hyperfine pattern, the sextet of the DMPO-C· radical and the DMPOX signal in a ratio of approximately 1:1:0.05 (Fig. 2e).

**Figure 1:**
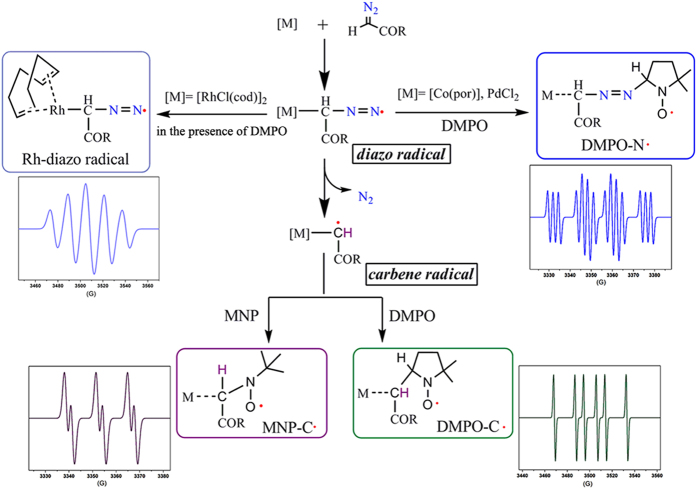
Proposed mechanism for the metal-mediated transformation of α-carbonyl diazomethanes into carbene radicals with diazo radicals as key intermediates and general EPR spectra of the detected radical species. Blue line: the quintet EPR signal of an Rh-diazo radical and the triplet-of-sextets EPR signal of a DMPO-trapped diazo radical (DMPO-N·); green line: the sextet EPR signal of a DMPO-trapped carbene radical (DMPO-C·); purple line: the doublet-of-triplets EPR signal of a MNP-trapped carbene radical (MNP-C·).

**Figure 2:**
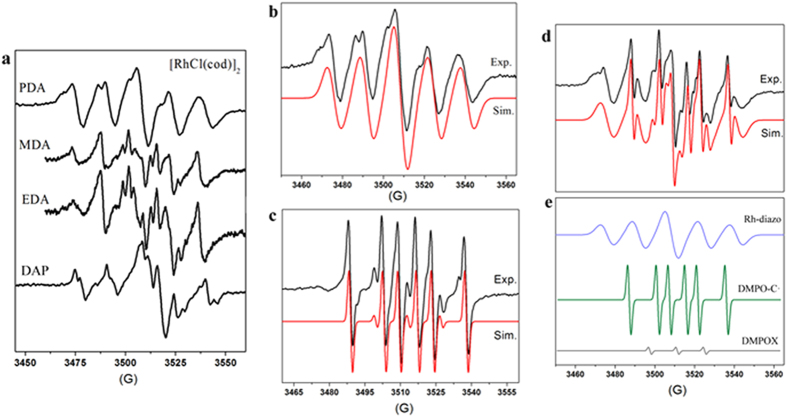
Quintet EPR signal of the dinitrogen radical and sextet EPR signal of the DMPO-trapped carbene radical (DMPO-C·). (**a**) Quintet EPR spectra obtained from the [RhCl(cod)]_2_-catalyzed PDA, MDA, EDA, and DAP reaction systems in the presence of DMPO. (**b**) Experimental (black line) and simulated (red line) quintet EPR spectra of the Rh-diazo radical obtained from the [RhCl(cod)]_2_-PDA system (detected at 1 min). (**c**) Sextet EPR spectrum of the DMPO-trapped carbene radical (DMPO-C·) obtained from the [RhCl(cod)]_2_-PDA system (detected at 30 min). (**d**) Multiple EPR spectra obtained from the [RhCl(cod)]_2_-PDA system (detected at 5 min), simulated as a mixture of Rh-diazo radical (blue line), DMPO-C· (green line) and DMPOX (gray line) in a ratio of approximately 1:1:0.05 (**e**).

Low temperature infrared and nuclear magnetic resonance spectroscopy have confirmed that the dominant steady-state species in the rhodium-catalyzed diazo compound reaction is the “Rh-diazoalkyl” adduct with a rhodium-carbon bond^7^. We assigned the unique quintet EPR signal to the unprecedented Rh-diazo radical, which consists of a diazo radical coordinated to an Rh center (Fig. 1). And the sextet of DMPO-C· was assigned to the DMPO-trapped C-centered carbene radical. Experimentally, the change of the quintet EPR signal into the sextet proceeded with N_2_ release, strongly suggesting that the Rh-carbene radical originates from the Rh-diazo radical.

**Figure 3:**
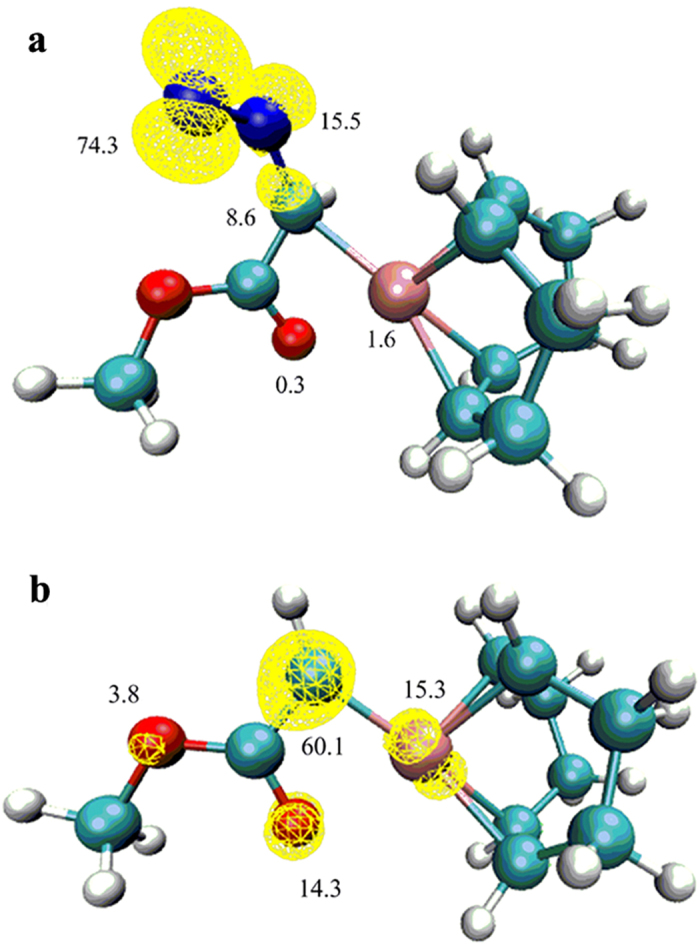
Calculated molecular structures and Mulliken atom spin densities for the Rh-diazo radical and Rh-carbene radical. (**a**) Rh-diazo radical; (**b**) Rh-carbene radical. The marked numbers refer to the calculated spin densities on the corresponding atoms.

DFT calculations were performed to probe the molecular structure and spin density distribution of the Rh-diazo radical (Fig. 3a). These calculations assign 97.2% of the spin density to the diazo moiety (70.8% on the terminal nitrogen atom, 17.9% on the central nitrogen atom and 8.5% on the carbon atom). The calculations indicate that the Rh-diazo radical could be an σ-state of diazo radical^24^. The smaller a^N^ should be attributed to the terminal^14^ N nucleus which has a typical “p-character” while the larger a^N^ should be assigned to the central^14^ N nucleus which has a substantial “s-character”. The calculated C-N-N angle of 123.7^o^ could also support the “σ-diazo” radical character. A spin density of only 2.3% was calculated at Rh (Supplementary Fig. S5a and Table S1). These results suggest that the [Rh(cod)] moiety is particularly suited to be an efficient stabilizing fragment for the diazo radical^30^. Experimentally, the detection of such Rh-diazo radical should be carried out in the presence of DMPO, indicating that the complex of Rh-diazo radical and DMPO must be more stable than the Rh-diazo radical itself, which is supported by its binding energy (−139.82 KJ mol^−1^) that is 35.62 KJ mol^−1^ lower than that of Rh-diazo radical (−104.2 KJ mol^−1^) (Supplementary Fig. S5b).

DFT calculations on the Rh-carbene radical (Fig. 3b) supported the EPR analyses that assigned a significant amount of spin density (60.1%) to the carbene carbon (Supplementary Fig. S6 and Table S2). Conversely, the spin density on the Rh center is predicted to be low (15.3%). Therefore, the Rh-carbene is better described as a carbon-centered Rh-carbene radical, which was efficiently captured by DMPO^19^.

Furthermore, although the Co-carbene radical generated by the [Co(3,5-Di^t^Bu-ChenPhyrin)]-EDA reaction has been detected by low-temperature EPR spectroscopy, a reaction system composed of [Co(por)] and EDA has been reported to be EPR silent^17^, suggesting that low-temperature EPR spectroscopy is not universally effective. Additionally, metal-catalyzed transformations of diazo compounds into carbene radicals are generally carried out at room temperature or higher; thus, high-temperature detection techniques are imperative to elucidating the transformation mechanisms. In this respect, the newly-developed spin-trapping method in conjunction with EPR offers a convenient method for the detection of radical intermediates under realistic reaction conditions.

### Triplet-of-sextets EPR signal of the DMPO-trapped diazo radical (DMPO-N·)

**Figure 4:**
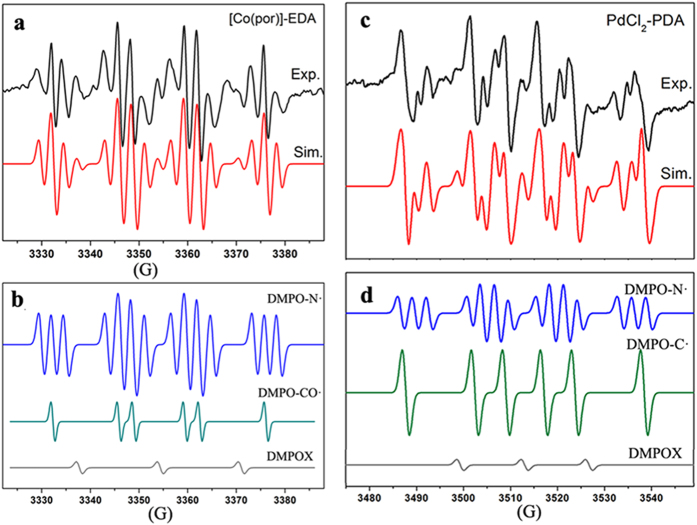
Triplet-of-sextets EPR signal of the DMPO-trapped diazo radical (DMPO-N·). (**a**) Experimental (black line) and simulated (red line) EPR spectra of the [Co(por)]-EDA system in the presence of DMPO, observed as a mixture of DMPO-N·, DMPO-CO· and DMPOX in a ratio of approximately 1:0.4:0.05 (**b);** (**c**) Experimental (black line) and simulated (red line) EPR spectra of the PdCl_2_-PDA system in the presence of DMPO, observed as a mixture of DMPO-N·, DMPO-C· and DMPOX in a ratio of approximately 0.9:1:0.05 (**d**).

We sought to detect the radicals generated in the [Co(por)]-EDA system in the presence of DMPO. The major signal observed (Fig. 4a) was a triplet-of-sextets (blue line in Fig. 4b) with HFCs of a^N^ = 13.58 G, a^H^ = 16.64 G, a^γ−N^ = 2.50 G; this signal was assigned to a DMPO-trapped N-centered radical adduct (DMPO-N·)^31^,^32^. The small γ-N hyperfine coupling constant clearly indicated the existence of the Co-diazo radical. The three-line DMPOX signal with a single nitrogen HFC of a^N^ = 16.50 G could also be observed (gray line in Fig. 4b). While a closer inspection of the spectrum (Fig. 4a) revealed the superposition by another DMPO-trapped carbon-centered radical, with HFCs of a^H^ = 16.64 G and a^N^ = 13.58 G (dark cyan in Fig. 4b; Supplementary Fig. S7). The HFCs values of this species are in the range of a DMPO-trapped CO-centered radical adduct^17^,^27^. The hyperfine pattern of Fig. 4a blurred when the reaction mixture was detected at a prolonged time.

DFT calculations on the Co-diazo radical (Supplementary Fig. S8a) assigned 10.8% of spin density on the terminal nitrogen atom, 2.9% on the central nitrogen atom and 97.6% on the Co atom while calculations on the transition state of Co-diazo radical (Supplementary Fig. S8b) assigned 63.1% of the spin density to the diazo moiety (26.0% on the terminal nitrogen atom, 4.3% on the central nitrogen atom and 32.8% on the carbon atom). These values are in consistent with the EPR analyses that the Co-diazo radical should be captured by DMPO.

Measuring the PdCl_2_-PDA system in the presence of DMPO yielded a composite EPR spectrum (Fig. 4c). After simulation, aside from the DMPOX signal (gray line in Fig. 4d, ratio of 0.05), the DMPO-trapped diazo radical adduct (DMPO-N·), with HFCs of a^N^ = 14.70 G, a^H^ = 17.30 G, a^γ−N^ = 3.00 G (blue line in Fig. 4d; ratio of 0.9), and the DMPO-trapped carbene radical adduct (DMPO-C·), with HFCs of a^N^ = 14.70 G, a^H^ = 21.30 G (green line in Fig. 4d; ratio of 1) was also identified (Supplementary Fig. S9). Measurements of the PdCl_2_-DAP system in the presence of DMPO, detected a ratio for the DMPO-N· signal (HFCs of a^N^ = 13.14 G, a^H^ = 16.48 G, a^γ−N^ = 2.64 G), DMPO-C· signal (HFCs of a^N^ = 14.27 G, a^H^ = 21.76 G) and DMPOX signal (HFC of a^N^ = 13.78 G) of 1:0.75:0.3 (Supplementary Fig. S10).

**Figure 5:**
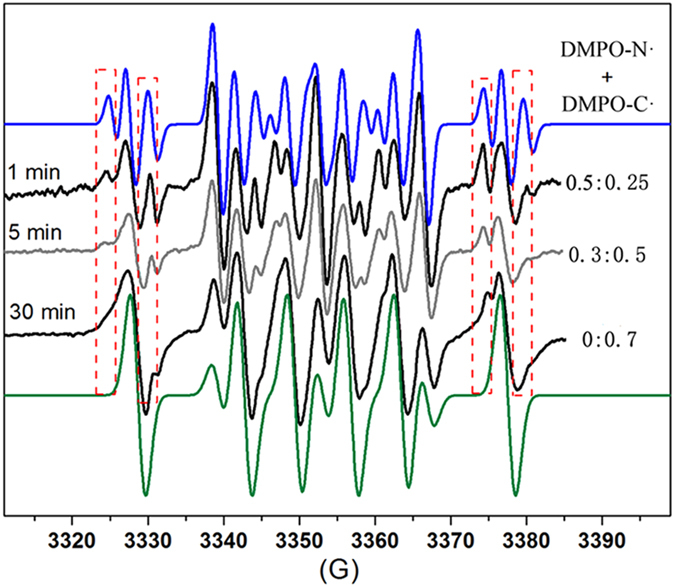
Time-dependent EPR spectra of the PdCl_2_-EDA system in the presence of DMPO. Over the course of 30 min, the ratio of DMPO-N· changes from 0.5 to 0, whereas the ratio of DMPO-C· changes from 0.25 to 0.7.

The DMPO-trapped carbene radical signal caused concentration-dependent changes to the EPR spectrum of the DMPO-trapped diazo radical^31^. Recording the EPR spectra of the PdCl_2_-EDA system in the presence of DMPO as a function of time allowed the observation of the gradual disappearance of the DMPO-N· signal (HFCs of a^N^ = 15.31 G, a^H^ = 19.74 G, a^γ−N^ = 2.56 G) along with an increase in the DMPO-C· signal (HFCs of a^N^ = 14.31 G, a^H^ = 21.04 G) (Fig. 5; Supplementary Fig. S11). At the highest concentration of the DMPO-trapped carbene radical, the predominant signals observed were the intense DMPO-C· sextet and the small three-line DMPOX signal (green line on Fig. 5). The disappearance of the DMPO-N· signal was also observed in the PdCl_2_-DAP system (Supplementary Fig. S12).

To further demonstrate that the C-centered radicals trapped by DMPO (DMPO-C·) are carbene radicals, MNP was used as the spin trap. Compared with the nitrone spin trap DMPO, the nitroso MNP spin trap is strongly electrophilic and sensitive to steric factors^33^,^34^,^35^. In particular, the β-hydrogen of the MNP-adduct is markedly dependent on the structure of the added radicals because they are directly attached to the MNP-nitrogen atom (Fig. 1).

### Doublet-of-triplets EPR signal of MNP-trapped carbene radical (MNP-C·)

**Figure 6:**
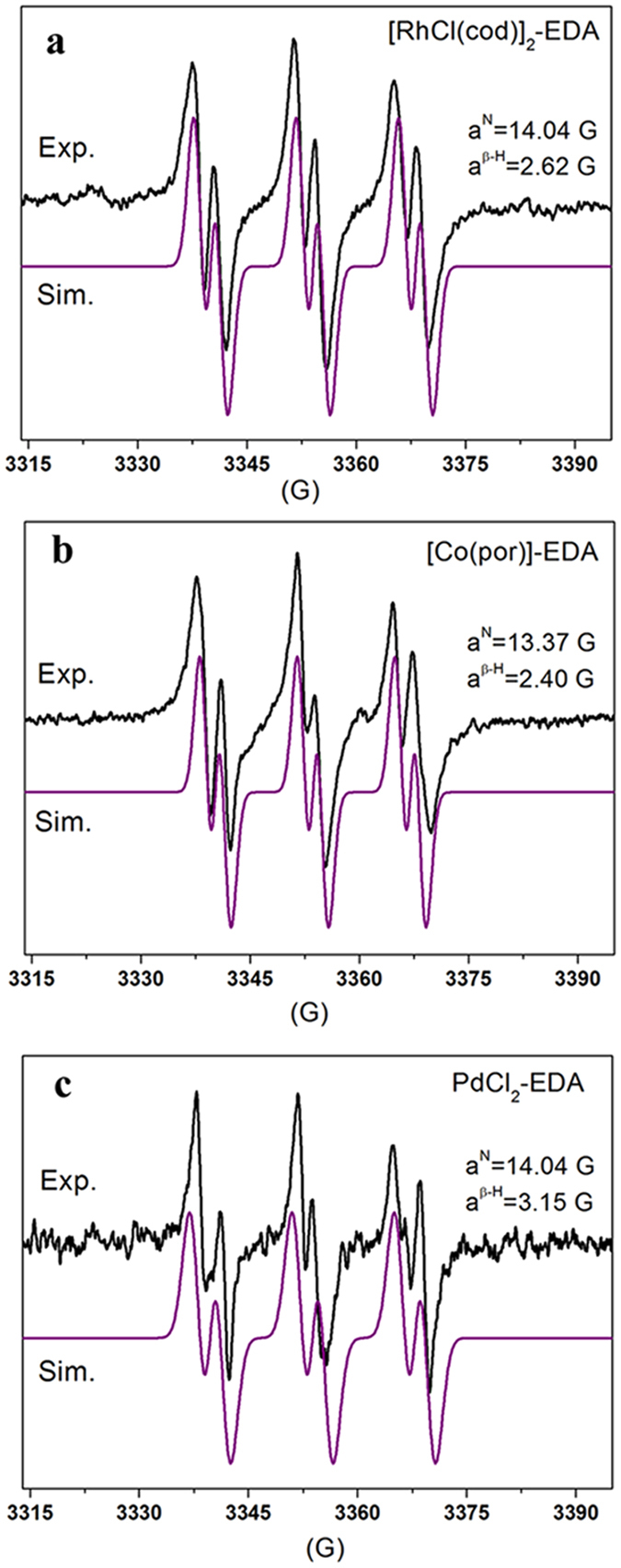
Doublet-of-triplets EPR signal of the MNP-trapped carbene radical (MNP-C∙). Experimental (black line) and simulated (purple line) EPR spectra of the [RhCl(cod)]_2_-EDA system (**a**), the [Co(por)]-EDA system (**b**) and the PdCl_2_-EDA system (**c**) in the presence of MNP.

Among all the [RhCl(cod)]_2_-, [Co(por)]- and PdCl_2_-catalyzed EDA systems tested in the presence of MNP, only the doublet-of-triplets EPR signal of the MNP-trapped C-centered radical (MNP-C·) was detected (Fig. 6). After simulation, the HFCs for the [RhCl(cod)]_2_-EDA system were observed to be a^N^ = 14.04 G and a^β−H^ = 2.62 G (1H) (Fig. 6a), whereas those for the [Co(por)]-EDA and PdCl_2_-EDA systems were a^N^ = 13.37 G and a^β−H^ = 2.40 G (1H) (Fig. 6b), and a^N^ = 14.04 G and a^β−H^ = 3.15 G (1H) (Fig. 6c), respectively. The small HFC of the β-H accounts for the highly distinctive doublet-of-triplets hyperfine pattern^33^, suggesting that the C-centered radicals trapped by MNP are definitely C-centered carbene radicals attached to a single proton. The deviation in the HFCs of the β-H units (2.62 G, 2.40 G and 3.15 G) is possibly due to the influence of the various metals bound to the carbene radicals. Because the π-system of the diazo radical is sufficiently large to allow for the approach of the N-O π-system of MNP, no MNP-trapped diazo radical adduct was detected. This observation clarifies that the C-centered radicals trapped by DMPO and MNP are metal-carbene radicals.

In conclusion, the Rh-, Co- and Pd-diazo radicals were confirmed in this work to be new members of the radical family and to be the active precursors of their corresponding metal-carbene radicals. The unique quintet EPR signal of a dinitrogen radical was recorded in the presence of DMPO at room temperature (RT) only in cases where α-carbonyl diazomethanes were complexed to [RhCl(cod)]_2_. DFT calculations on the Rh-diazo radical indicated dominant diazo radical character, with 97.2% of the spin density distributed on the diazo moiety, which was stabilized to be detectable by DMPO (Supplementary Fig. S20). In contrast, the Co- and Pd-diazo radicals were EPR silent at RT, but were dynamically captured by DMPO (Supplementary Fig. S21) to form spin adducts (DMPO-N·) that show a triplet-of-sextets EPR signal.

The spin-trapping combined with RT-EPR spectroscopy was also observed to provide a powerful tool for the detection of metal-carbene radicals. The EPR spectrum of the DMPO-carbene adduct (DMPO-C·) showed a sextet signal and MNP-carbene adduct (MNP-C·) showed a highly distinctive doublet-of-triplets. In this way, metal-diazo radical signals were easily distinguished from metal-carbene radical signals. In addition, the different β-hydrogen hyperfine coupling constants of the MNP adducts with Rh-, Co- and Pd-carbene radicals illustrated the various effects of the metal centers on the spin-density distributions of metal-carbene radicals.

Experimentally, the transformation of the diazo radical signal into signals indicative of DMPO-carbene radical adducts with simultaneous N_2_ elimination demonstrated that metal-carbene radicals originated from metal-diazo radicals. Thus, the transformation of α-carbonyl diazomethane into a metal-carbene radical was confirmed to be a two-step process with a metal-diazo radical as the key intermediate. We hope these new findings will contribute to the understanding of the fundamental reactivity of metal-activated α-carbonyl diazomethane reaction systems and allow for the discovery of new reactions involving metal-diazo and metal-carbene radicals.

## Methods

### Materials

5,5-dimethyl-1-pyrroline-N-oxide (DMPO, 98%, Adamas Reagent Co. Ltd) and 2-methyl-2-nitrosopropane (MNP, 98%, Adamas Reagent Co. Ltd) were stored at −20 °C and used as received. The solvents were purchased from Sinopharm Chemical Reagent Co. Ltd and distilled before use.

### Analytic Instrumentation

X-band EPR spectra were recorded on Bruker Biospin A200 spectrometer and Bruker A300-6/1. Detection conditions: Sweep Width: 100 -200 G; Microwave Frequency: 9.4-9.8 GHz; Microwave Power: 1.9-20 mW; Modulation amplitude of 1.00 G; Modulation Frequency: 100.00 kHz. The spectra were simulated by iteration of the anisotropic g-values, hyperfine coupling constants and line widths using biomolecular EPR spectroscopy software developed by W. R. Hagen.

### RT EPR spectroscopy in conjunction with spin-trapping technique

After strict extrusion of O_2_, a solution of toluene (5 ml), spin traps (10 μL of DMPO or 10 mg of MNP) and metal catalysts (30 mg) were stirred under N_2 _at 60 ^o^C, α-carbonyl diazomethanes (500 μL or 50 mg) were introduced to the systems. Then 2 μL of the solution was collected at different time and detected by EPR at room temperature.

### DFT calculations

BP86 density functional method^36^,^37^ in combination with def2-SVP basis-set was used to optimize the geometry of all systems. The basis-set used was upgraded to def2-TZVP^38^ when evaluating single-point energies. The calculations were conducted with ORCA 3.0.3 program. Spin density and spin population were calculated by Multiwfn 3.3.7 program *via* Mulliken method^39^.

## Additional Information

**How to cite this article**: Li, F. *et al.* Metal-Diazo Radicals of a-Carbonyl Diazomethanes. *Sci. Rep.* **6**, 22876; doi: 10.1038/srep22876 (2016).

## Supplementary Material

Supplementary Information
