# Supplementary material for: Metal-Diazo Radicals of α-Carbonyl Diazomethanes
Source: Sci Rep. 2016 Mar 10;6:22876. doi: 10.1038/srep22876 (PMC4785408; doi:10.1038/srep22876)
Supplement: Supplementary Information [file srep22876-s1.pdf]

Supplementary Information for

Metal-Diazo Radicals of α-Carbonyl Diazomethanes

Feifei Li, Longqiang Xiao & Lijian Liu*

Department of Polymer Science, College of Chemistry and Molecular Sciences, Wuhan University, Wuhan 430072, China

*To whom correspondence should be addressed. E-mail: liulj@whu.edu.cn

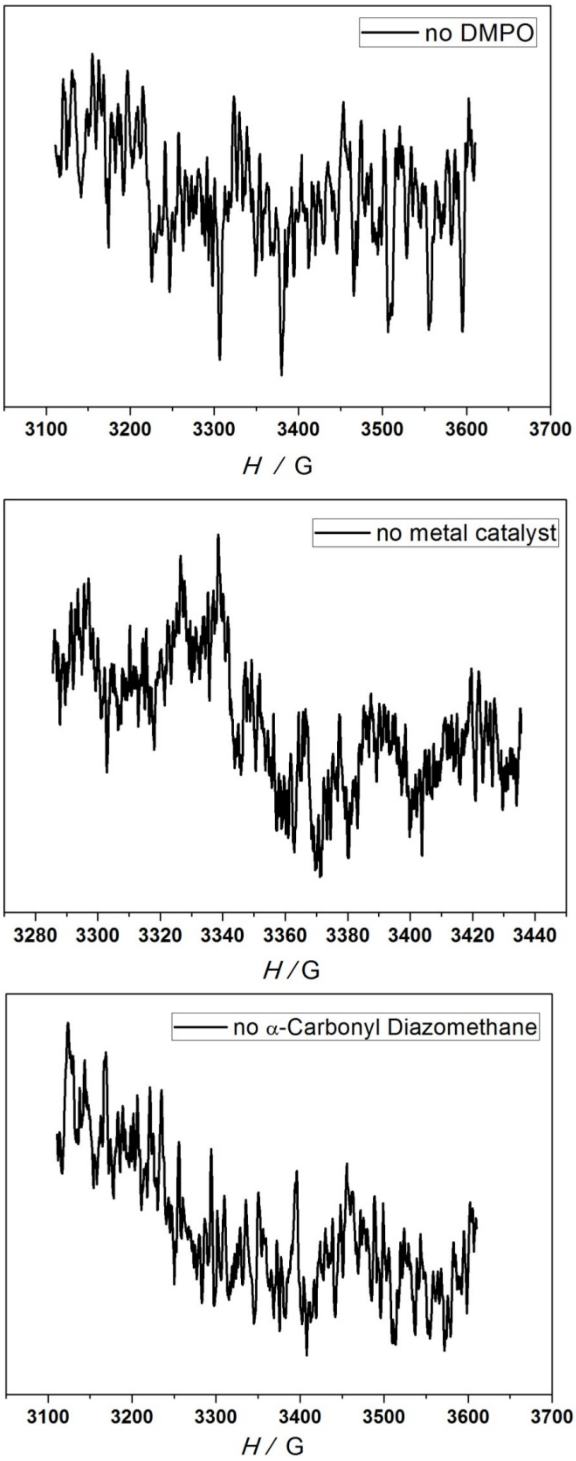

Supplementary Figure 1. Control experiments suggest that in the absence of metal catalysts, α-carbonyl diazomethane and DMPO, no EPR signals could be detected, which confirms that the radicals are generated during the interaction of the metal-catalysts and the diazocompounds.

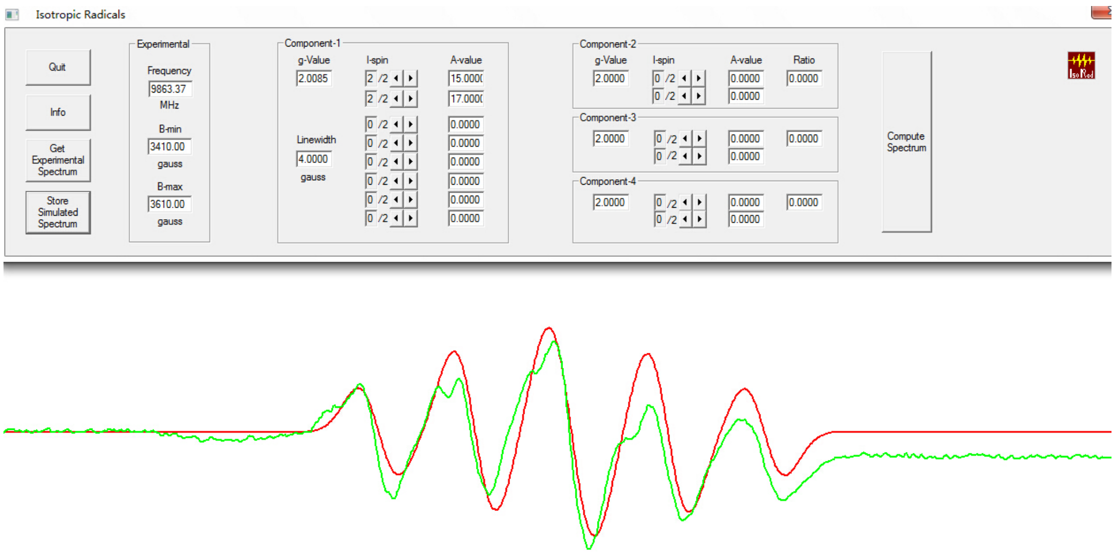

Supplementary Figure 2A. Experimental (green line) and simulated (red line) EPR spectra obtained from the $[RhCl(cod)]_2$-PDA system (detected at 1 min).

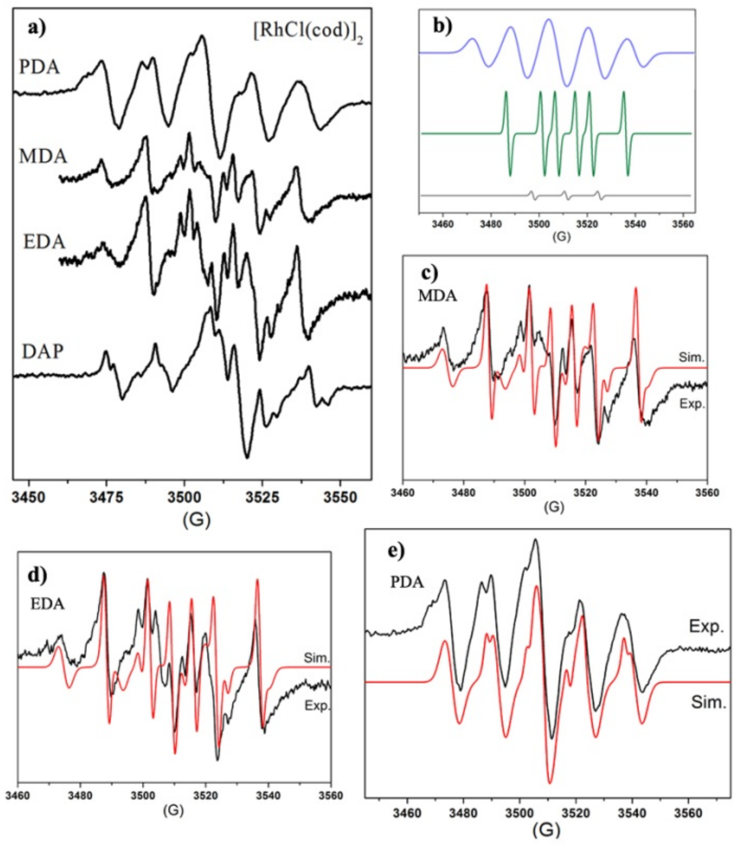

Supplementary Figure 2B. **a**, Quintet EPR spectra obtained from the $[RhCl(cod)]_2$-catalyzed PDA, MDA, EDA, and DAP reaction systems. **b**, quintet EPR signal (blue line), sextet EPR signal (green line) and triplet EPR signal (gray line). **c**, Experimental (black line) and simulated (red line) EPR spectra of the $[RhCl(cod)]_2$-catalyzed MDA system. **d**, Experimental (black line) and simulated (red line) EPR spectra of the $[RhCl(cod)]_2$-catalyzed EDA system. **e**, Experimental (black line) and simulated (red line) EPR spectra of the $[RhCl(cod)]_2$-catalyzed PDA system.

Analysis:

The EPR spectra shown in Figure 2a displays more splitting besides the quintet signal, partly due to the radical motion in solvent, and more importantly as a consequence of the superposition by other paramagnetic species in low concentration. These spectra are further simulated (red line) as a combination of the quintet hyperfine pattern (blue line), the sextet of the DMPO-C· radical (green line) and the triplet DMPOX signal (Figure. b). In the case of $[RhCl(cod)]_2$-PDA system, the experimental spectrum could be accurately simulated as a mixture of Rh-diazo radical, DMPO-C· and DMPOX in a ratio of approximately 10:1:0.1 (Figure. e). In the case of $[RhCl(cod)]_2$-MDA system (Figure. c) and $[RhCl(cod)]_2$-MDA system (Figure. d), the ratio of Rh-diazo radical, DMPO-C· and DMPOX is approximately 1:1:0.1. In the case of $[RhCl(cod)]_2$-DAP system, while the quintet signal is strong, the DMPO-C· and DMPOX signals cannot be explicitly distinguished.

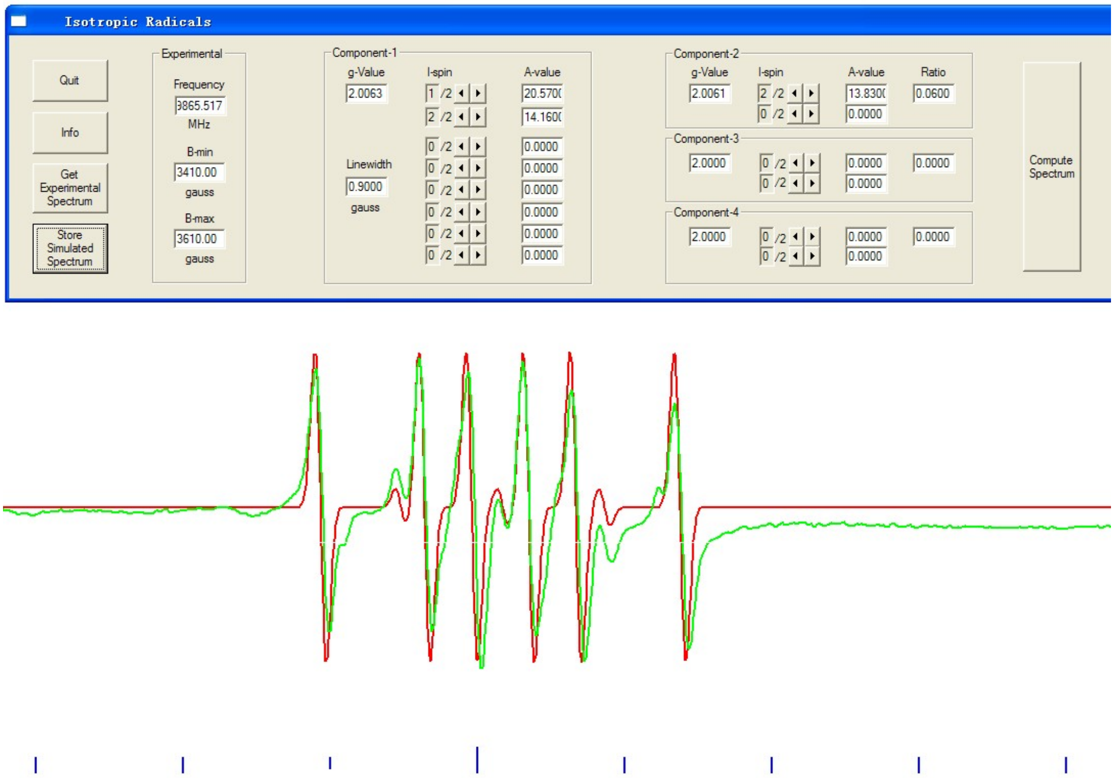

Supplementary Figure 3. Experimental (green line) and simulated (red line) EPR spectra of the DMPO-trapped carbene radical (DMPO-C·) and the DMPOX signal.

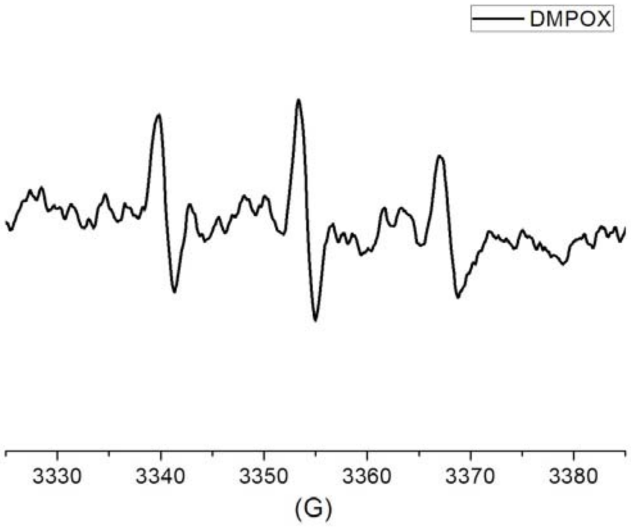

Supplementary Figure 4. EPR spectrum of DMPOX detected with the scandium triflate system ([Sc(oTf)]-EDA) in the presence of DMPO. Only the three-line DMPOX signal could be detected when no EDA-participated reactions occurred in the presence of [Sc(oTf)].

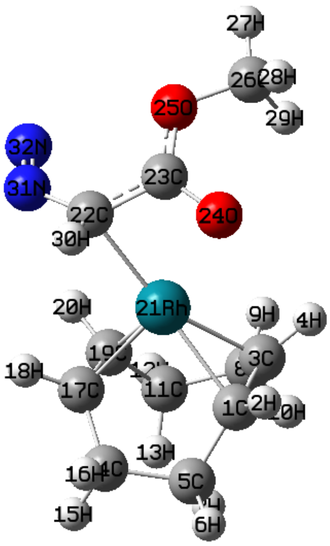

Supplementary Figure 5a. Calculated molecular structure for possible Rh-diazo radical (the binding energy is calculated to be -104.2 KJ mol^{-1}).

Supplementary Table S1. Spin-density distribution for Rh-diazo radical

Atom	Alpha_pop.	Beta_pop.	Spin_pop.	Atomic charge
1(C)	3.02500	3.02670	-0.00170	-0.05170
2(H)	0.47105	0.47098	0.00006	0.05797
3(C)	3.04001	3.04078	-0.00078	-0.08079
4(H)	0.47274	0.47255	0.00018	0.05471

5(C)	2.97331	2.97311	0.00020	0.05357
6(H)	0.48851	0.48854	-0.00003	0.02295
7(H)	0.49082	0.49086	-0.00004	0.01831
8(C)	2.95855	2.95836	0.00019	0.08310
9(H)	0.48293	0.48307	-0.00014	0.03400
10(H)	0.48566	0.48570	-0.00004	0.02864
11(C)	2.96813	2.96812	0.00001	0.06376
12(H)	0.48704	0.48703	0.00001	0.02593
13(H)	0.49059	0.49059	0.00001	0.01882
14(C)	2.95704	2.95712	-0.00007	0.08584
15(H)	0.48687	0.48685	0.00002	0.02629
16(H)	0.48204	0.48207	-0.00003	0.03589
17(C)	3.04626	3.04753	-0.00127	-0.09379
18(H)	0.46906	0.46877	0.00029	0.06217
19(C)	3.04920	3.04854	0.00066	-0.09774
20(H)	0.46598	0.46816	-0.00217	0.06586
21(Rh)	8.71558	8.69288	0.02269	-0.40846
22(C)	2.99459	2.90983	0.08476	0.09558
23(C)	2.91680	2.90889	0.00791	0.17432
24(O)	4.13543	4.12986	0.00558	-0.26529
25(O)	4.10688	4.10721	-0.00033	-0.21410
26(C)	2.95150	2.94900	0.00250	0.09950
27(H)	0.46775	0.46766	0.00009	0.06460
28(H)	0.46846	0.46864	-0.00018	0.06290
29(H)	0.46806	0.46842	-0.00035	0.06352
30(H)	0.46004	0.46513	-0.00509	0.07483
31(N)	3.60154	3.42268	0.17886	-0.02422
32(N)	3.92259	3.21439	0.70819	-0.13698
		Total spin electrons:	1.00000	

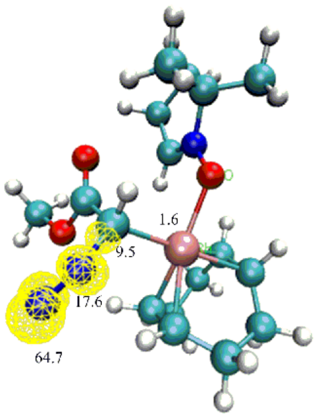

Supplementary Figure 5b. Calculated molecular structure for possible DMPO-Rh-diazo radical complex (the binding energy is calculated to be -139.82 KJ mol^{-1}).

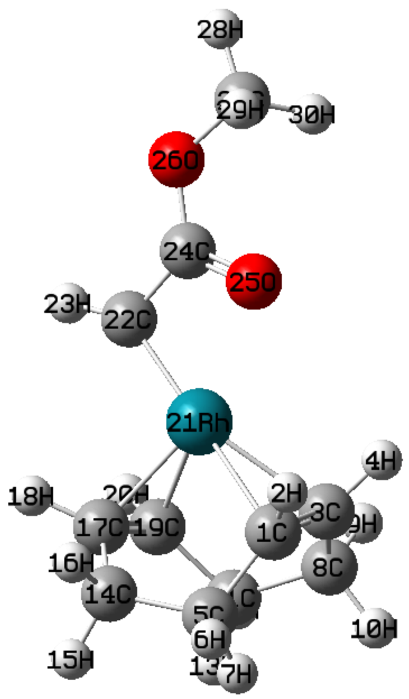

Supplementary Figure 6. Calculated molecular structure for Rh-carbene radical.

Supplementary Table S2. Spin-density distribution for Rh-carbene radical

Atom	Alpha_pop.	Beta_pop.	Spin_pop.	Atomic charge
1(C)	3.05895	3.00432	0.05464	-0.06327
2(H)	0.47437	0.47421	0.00016	0.05142
3(C)	3.06031	3.03474	0.02557	-0.09505
4(H)	0.47260	0.47148	0.00112	0.05591
5(C)	2.97227	2.97724	-0.00497	0.05049
6(H)	0.49008	0.48791	0.00216	0.02201
7(H)	0.49171	0.49092	0.00078	0.01737
8(C)	2.95799	2.96075	-0.00276	0.08127
9(H)	0.48207	0.48203	0.00004	0.03590
10(H)	0.48862	0.48366	0.00496	0.02771
11(C)	2.97018	2.96867	0.00151	0.06115
12(H)	0.49016	0.49023	-0.00007	0.01962
13(H)	0.49179	0.49187	-0.00008	0.01633
14(C)	2.96188	2.95777	0.00412	0.08035
15(H)	0.48969	0.48737	0.00231	0.02294
16(H)	0.48136	0.48167	-0.00032	0.03697
17(C)	3.03870	3.04986	-0.01117	-0.08856
18(H)	0.47465	0.47438	0.00027	0.05097
19(C)	3.04072	3.04830	-0.00758	-0.08901
20(H)	0.47475	0.47373	0.00102	0.05151
21(Rh)	8.70845	8.55587	0.15259	-0.26432
22(C)	3.33535	2.73383	0.60151	-0.06918

23(H)	0.47927	0.50452	-0.02525	0.01621
24(C)	2.88609	2.87089	0.01519	0.24302
25(O)	4.21728	4.07416	0.14311	-0.29144
26(O)	4.14296	4.10458	0.03838	-0.24754
27(C)	2.94751	2.95030	-0.00279	0.10219
28(H)	0.47336	0.47338	-0.00001	0.05326
29(H)	0.47322	0.47047	0.00275	0.05631
30(H)	0.47366	0.47087	0.00279	0.05547

Total spin electrons: 1.00000

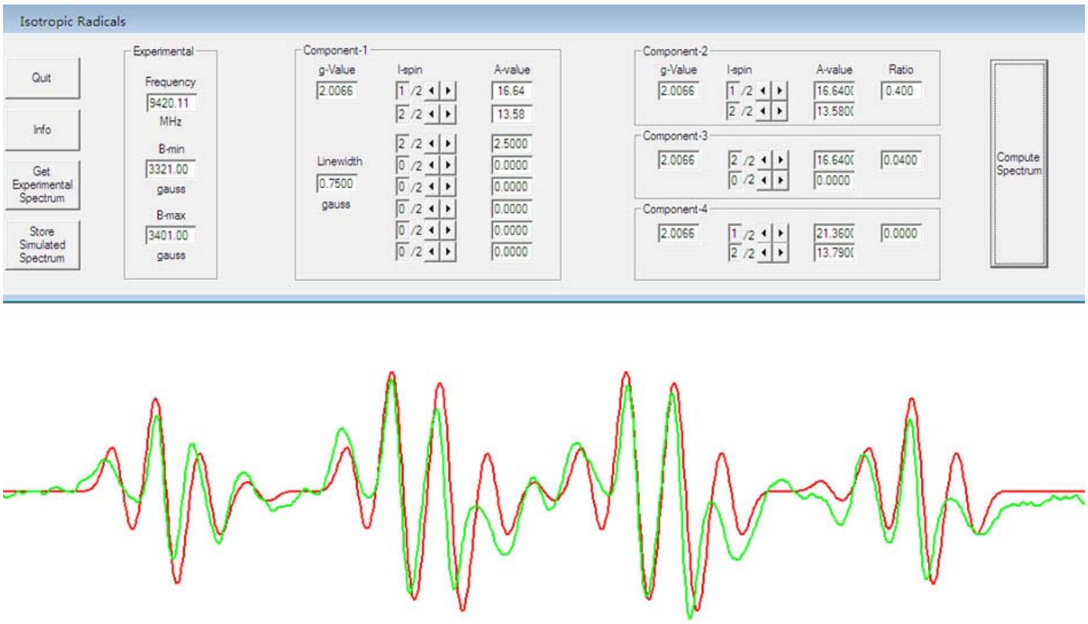

Supplementary Figure 7. Experimental (green line) and simulated (red line) EPR spectra of the [Co(por)]-EDA system in the presence of DMPO, observed as a mixture of DMPO-N·, DMPO-CO· and DMPOX in a ratio of approximately 1:0.4:0.04.

Analysis:

We calculated the N_2 release process from Co-diazo radical to Co-carbene radical and obtained a transition state of Co-diazo radical, which holds higher energy level (+61.02 KJ/mol). DFT calculations assigned 63.1% of the spin density to the diazo moiety (26.0% on the terminal nitrogen atom, 4.3% on the central nitrogen atom and 32.8% on the carbon atom, reported in the manuscript). The results clearly proved that spin transfer process occurred from Co center to the diazo moiety.

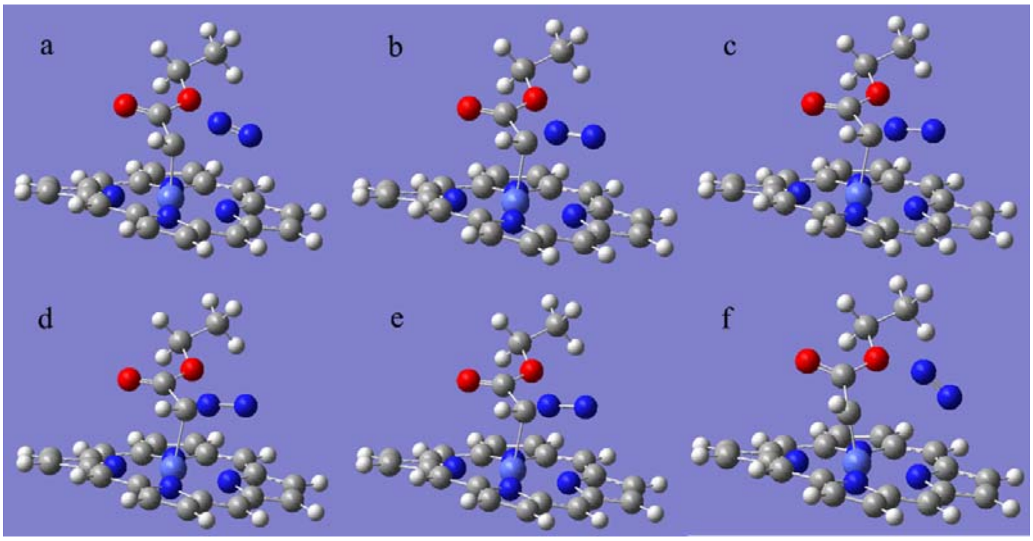

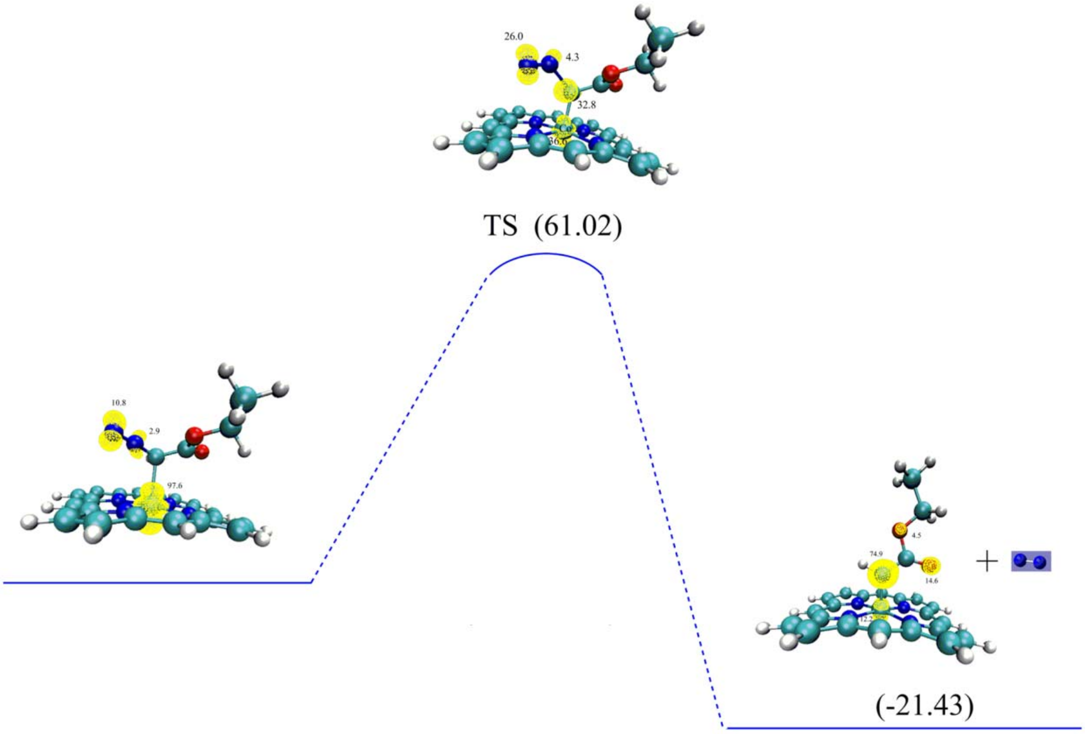

Supplementary Figure 8a. Calculation on the N_2 release process from Co-diazo adduct to Co-carbene radical via the transition state of Co-diazo radical.

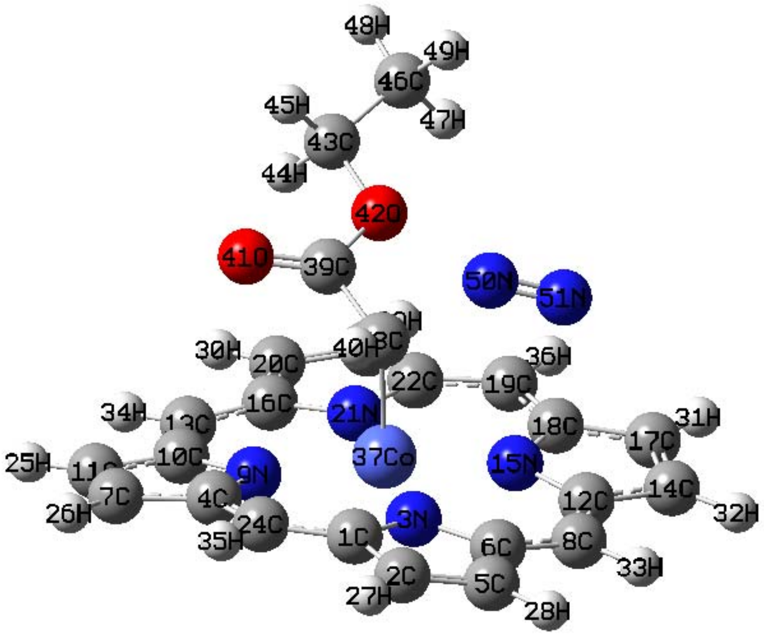

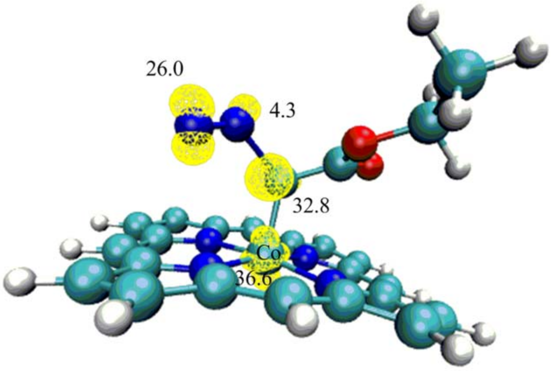

Supplementary Figure 8b. Calculated molecular structure for the transition state of Co-diazo radical.

Supplementary Table S3. Spin-density distribution for the transition state of Co-diazo radical.

Alpha_pop.	Beta_pop.	Spin_pop.	Atomic	charge
1(C)	2.94589	2.94874	-0.00285	0.10537
2(C)	3.00954	3.00883	0.00071	-0.01836
3(N)	3.59808	3.59934	-0.00126	-0.19742
4(C)	2.94018	2.94152	-0.00134	0.11830
5(C)	3.00922	3.01093	-0.00171	-0.02016
6(C)	2.93994	2.94216	-0.00222	0.11790
7(C)	3.00752	3.00735	0.00017	-0.01487
8(C)	3.03946	3.03050	0.00896	-0.06996

9(N)	3.59202	3.60552	-0.01350	-0.19754
10(C)	2.93018	2.93119	-0.00101	0.13863
11(C)	3.00662	3.00665	-0.00003	-0.01327
12(C)	2.93836	2.94025	-0.00189	0.12139
13(C)	3.02680	3.03180	-0.00501	-0.05860
14(C)	3.00489	3.00599	-0.00110	-0.01088
15(N)	3.60554	3.59722	0.00832	-0.20275
16(C)	2.94030	2.94155	-0.00125	0.11815
17(C)	3.00611	3.00733	-0.00122	-0.01343
18(C)	2.93991	2.94289	-0.00298	0.11720
19(C)	3.03510	3.02961	0.00549	-0.06471
20(C)	3.01056	3.00918	0.00138	-0.01974
21(N)	3.60271	3.60969	-0.00698	-0.21240
22(C)	2.93569	2.93649	-0.00080	0.12782
23(C)	3.01277	3.01446	-0.00170	-0.02723
24(C)	3.03335	3.03390	-0.00055	-0.06725
25(H)	0.50065	0.50075	-0.00009	-0.00140
26(H)	0.50068	0.50080	-0.00012	-0.00148
27(H)	0.50078	0.50083	-0.00005	-0.00161
28(H)	0.50045	0.50039	0.00006	-0.00083
29(H)	0.50117	0.50116	0.00000	-0.00233
30(H)	0.50098	0.50106	-0.00007	-0.00204
31(H)	0.50150	0.50125	0.00026	-0.00275
32(H)	0.50144	0.50115	0.00029	-0.00260
33(H)	0.50239	0.50286	-0.00047	-0.00525
34(H)	0.50389	0.50368	0.00021	-0.00757
35(H)	0.50243	0.50241	0.00003	-0.00484
36(H)	0.50314	0.50341	-0.00027	-0.00654
37(Co)	13.52791	13.16183	0.36608	0.31026
38(C)	3.13411	2.80583	0.32828	0.06006
39(C)	2.92311	2.92603	-0.00292	0.15087
40(H)	0.47075	0.47404	-0.00329	0.05522
41(O)	4.11760	4.08631	0.03129	-0.20391
42(O)	4.13317	4.13370	-0.00053	-0.26688
43(C)	2.94329	2.94385	-0.00057	0.11286
44(H)	0.47890	0.47893	-0.00003	0.04216
45(H)	0.47996	0.47942	0.00054	0.04063
46(C)	3.03433	3.03406	0.00026	-0.06839
47(H)	0.47746	0.47746	-0.00001	0.04508
48(H)	0.48191	0.48197	-0.00005	0.03612
49(H)	0.47579	0.47582	-0.00003	0.04839
50(N)	3.53646	3.49334	0.04312	-0.02981
51(N)	3.65501	3.39457	0.26044	-0.04959

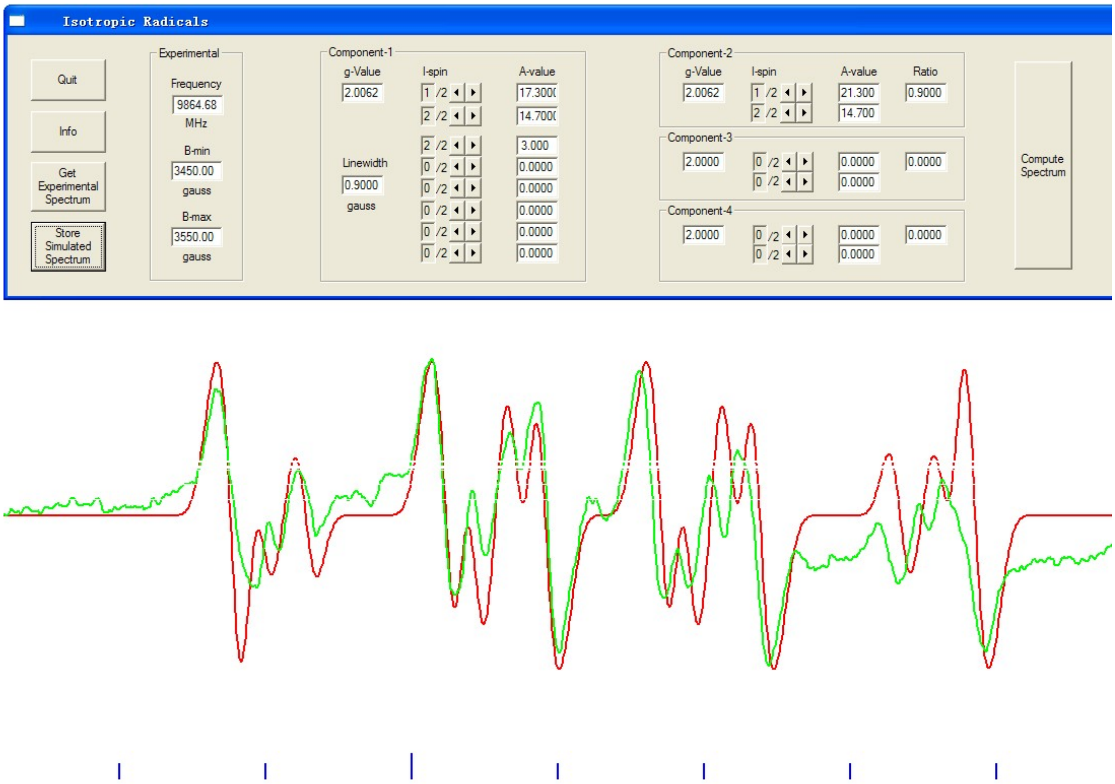

Supplementary Figure 9. Experimental (green line) and simulated (red line) EPR spectra of the $PdCl_2$-PDA system in the presence of DMPO, observed as a mixture of DMPO-N·, DMPO-C· and DMPOX in a ratio of approximately 0.9:1:0.03.

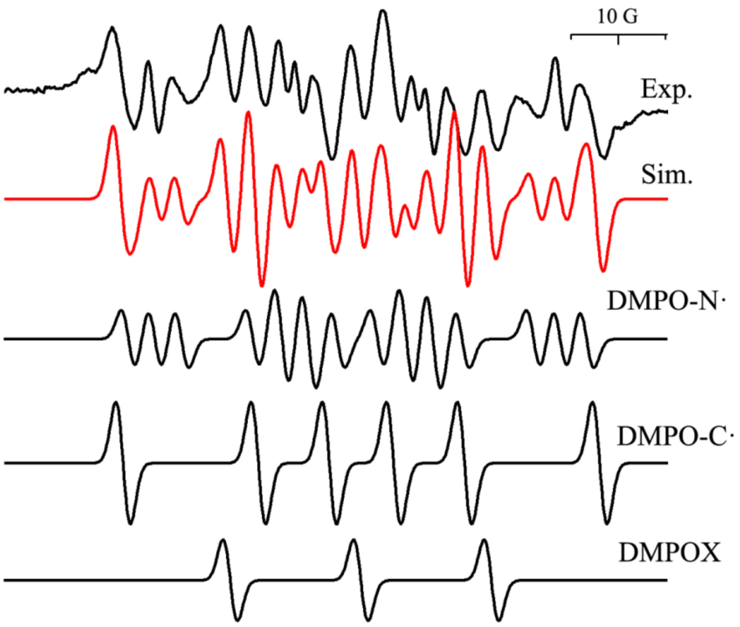

Supplementary Figure 10. EPR spectra of $PdCl_2$-diazoacetophenone reaction (DAP) system detected 1 min in the presence of DMPO (experimental (black line) and simulated (red line)). The EPR spectrum was simulated as a mixture of DMPO-N· (HFCs of a^N=13.14 G, a^H=16.48 G, $a^{\gamma\text{-}N}$=2.64 G), DMPO-C·(HFCs of a^N=14.27 G, a^H=21.76 G) and DMPOX (HFC of a^N=13.78 G) in a ratio of approximately 1:0.75:0.3.

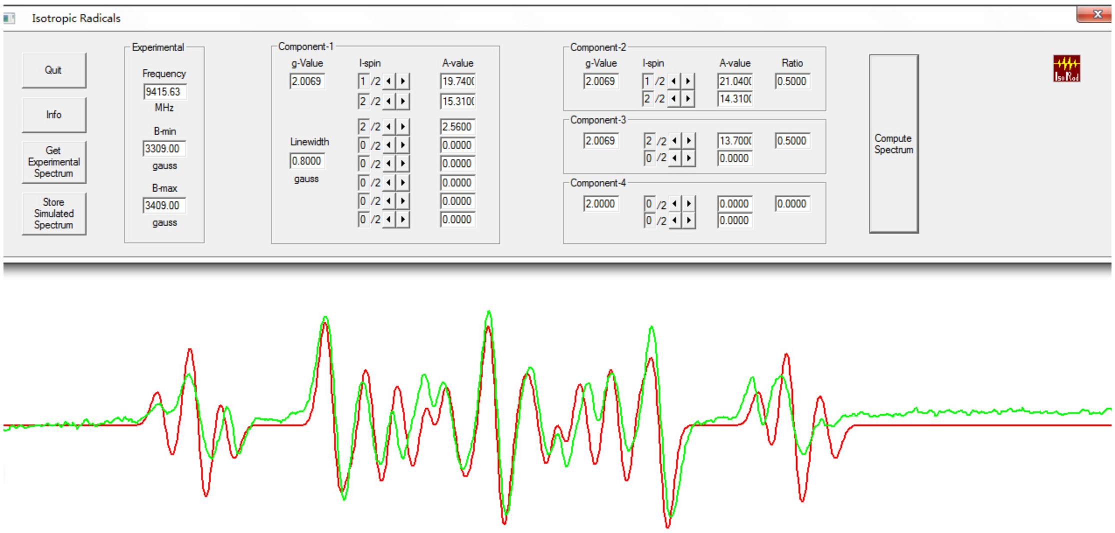

Supplementary Figure 11. Experimental (green line) and simulated (red line) EPR spectra of the $PdCl_2$-EDA system detected 1 min in the presence of DMPO, observed as a mixture of DMPO-N·, DMPO-C· and DMPOX in a ratio of approximately 1:0.5:0.5 ratio.

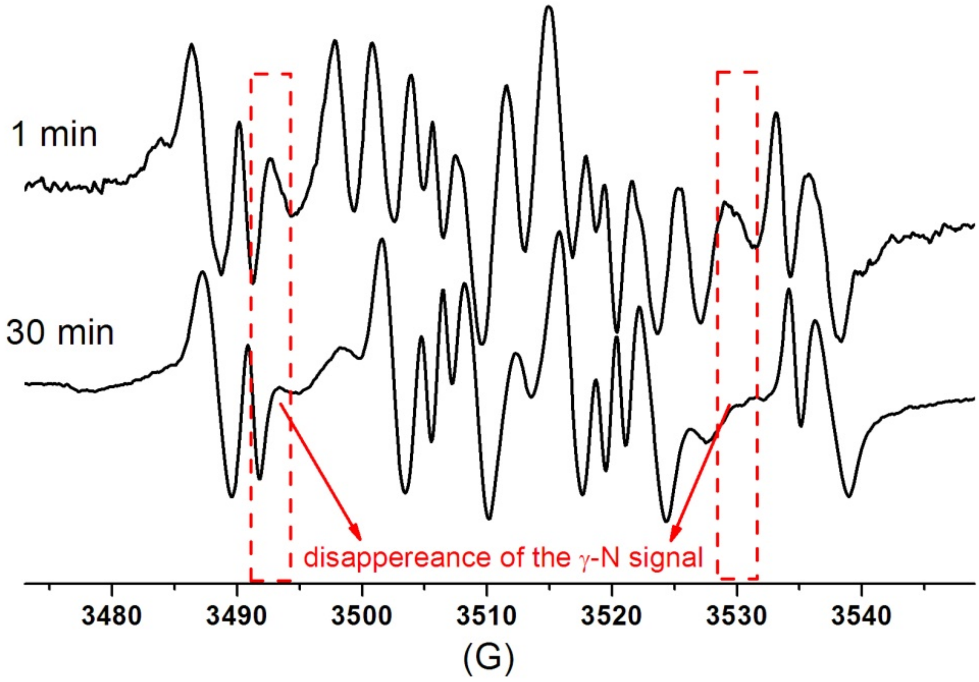

Supplementary Figure 12. EPR spectra of the reaction system of the $PdCl_2$-diazoacetophenone in the presence of DMPO detected at 1 min and 30 min. The disappearance of the typical γ-N signal of the DMPO trapped diazo radical adduct (DMPO-N·) could be clearly observed.

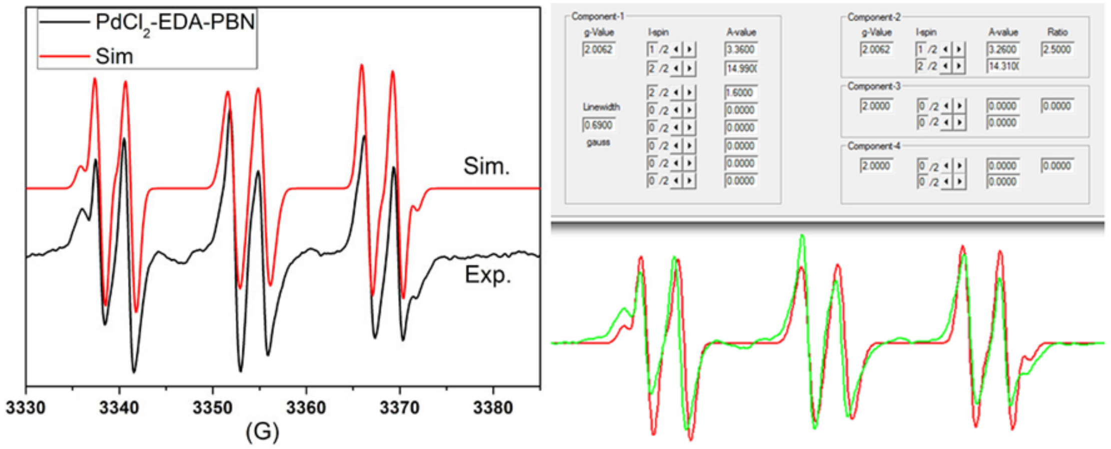

Supplementary Figure 13. EPR spectra of the reaction system of the $PdCl_2$-PDA in the presence of PBN (the green line: the experimental line; the red line: the simulation line).

Analysis: Besides DMPO and MNP, *N-tert*-butyl-α-phenylnitrone (PBN) was also used as spin trap to trap the radical species.

To $PdCl_2$-PDA system in the presence of PBN, the hyperfine coupling constants (HFCs) of a^N=14.99 G, a^H=3.36 G, $a^{\gamma\text{-}N}$=1.60 G for a trapped N-centered radical (PBN-N·), the HFCs of a^N=14.31 G, a^H=3.26 G for a trapped C-centered radical (PBN-C·).

Supplementary Table S4: g-values of the detected radicals:

Reaction system	Radical	g-value
	Rh-diazo radical	2.0085
$[RhCl(cod)]_2$-catalyzed system	DMPO-C·	2.0063
	DMPOX	2.0063
[Sc(oTf)]-EDA system	DMPOX	2.0069
	DMPO-N·	2.0066
[Co(por)]-EDA system	DMPO-C·	2.0066
	DMPOX	2.0066
	DMPO-N·	2.0062
$PdCl_2$-PDA system	DMPO-C·	2.0062
	DMPOX	2.0062

Analysis:

To exclude the possibility that the detected signals may stem from minor paramagnetic impurities, spin counting was performed (double integration of the detected signals referenced against a

reference compound). DPPH (as the reference compound; $1.5*10^{-2}$ mol/L) and Rh-diazo radical detected in the $[RhCl(cod)]_2$-PDA system (the catalyst concentration is about $1.2*10^{-2}$ mol/L). The details of the detection parameters:

For DPPH (supplementary Fig. 14): Center Field 3359.84 G; Static Field 3319.86 G; Microwave Frequency 9.417 GHz; Microwave power 19.12 mW; Receiver Gain $1.00*10^3$; Modulation Frequency 100.00 KHz; Modulation Amplitude 0.50 G.

For Rh-diazo radical (supplementary Fig. 16): Center Field 3510.00 G; Static Field 3410.00 G; Microwave Frequency 9.863 GHz; Microwave power 20.08 mW; Receiver Gain $1.00*10^5$; Modulation Frequency 100.00 KHz; Modulation Amplitude 1.00 G.

The Bruker WinEPR (software) was used to double integration of the signal (see below). Although the signal-to-noise of the quintet signal observed with Rh-diazo radical is weak than that of DPPH reference sample, the absolute signal intensity of Rh-diazo radical is lower than that of DPPH, since DPPH is a stable standard –NN▪ radical while Rh-diazo radical is an active radical intermediate that cannot be presented in high amount. Besides, we also traced the gradual disappearance of the Rh-diazo radical and DMPO-N· signals along with an increase in the DMPO-C· signal. Taking the two points into account, we are convinced that the detected signals cannot be stem from minor paramagnetic impurities.

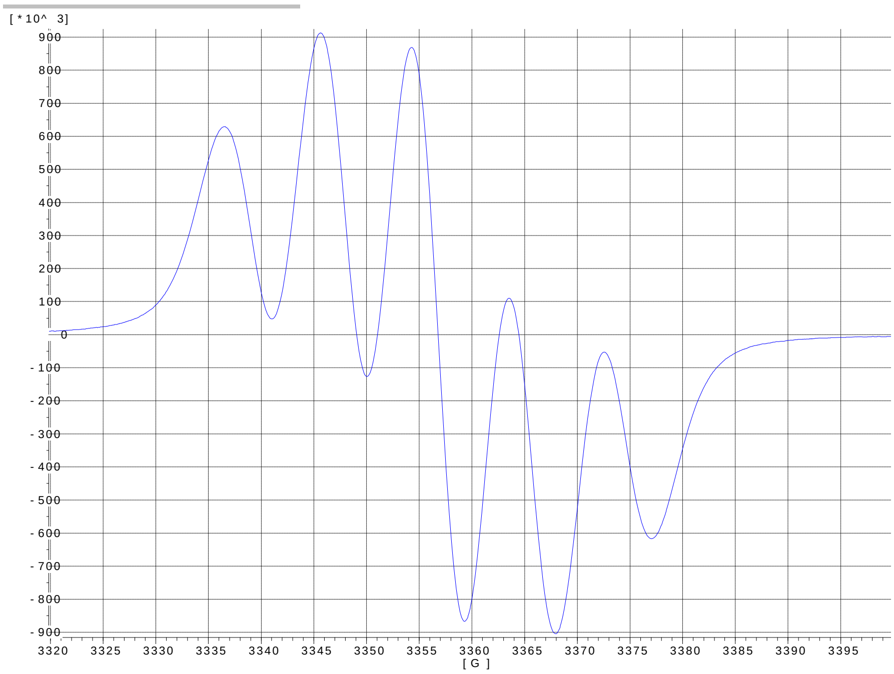

Supplementary Figure 14. EPR spectrum of DPPH.

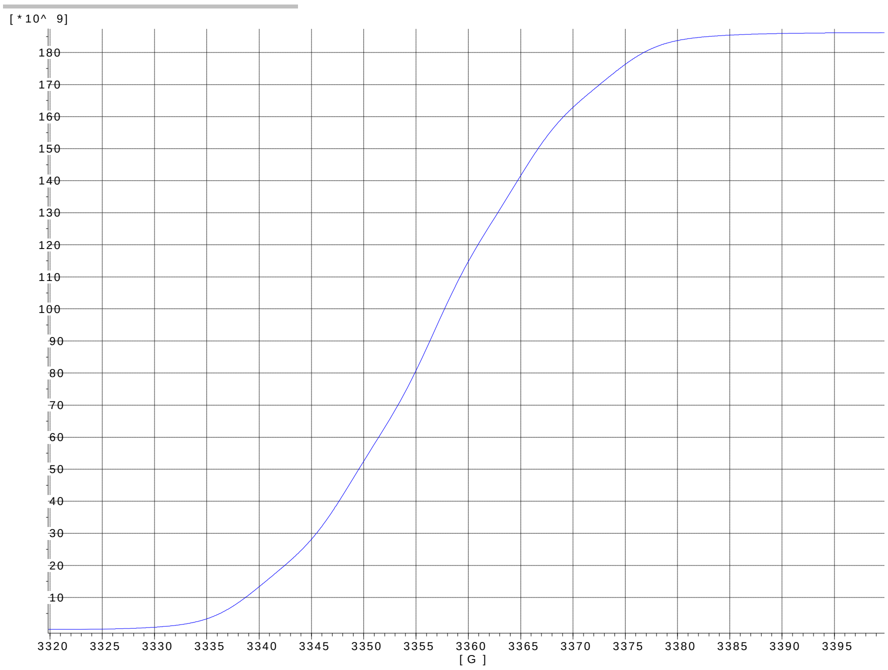

Supplementary Figure 15. Double integrate of DPPH signal.

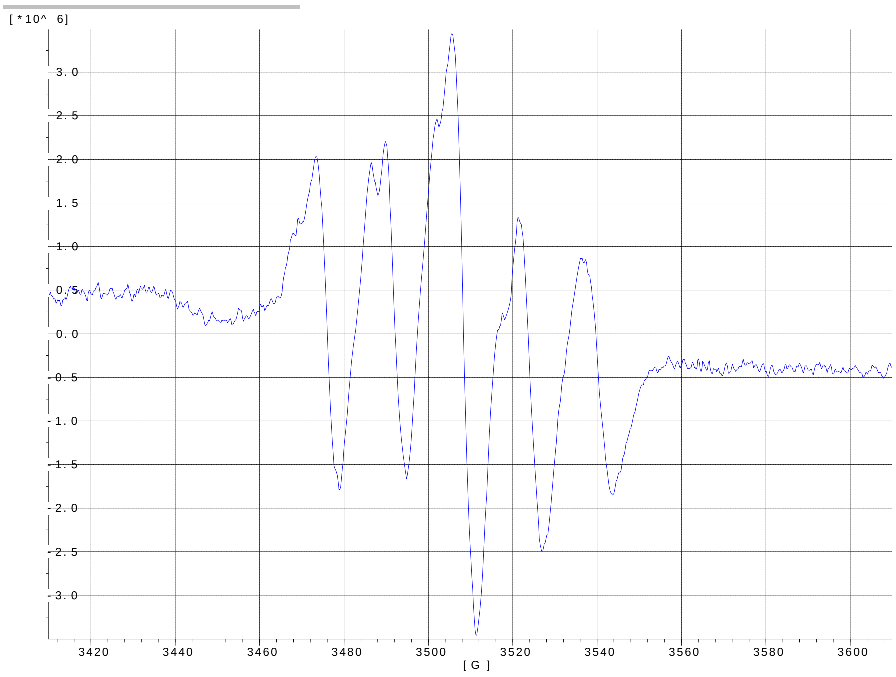

Supplementary Figure 16. EPR spectrum of Rh-diazo radical.

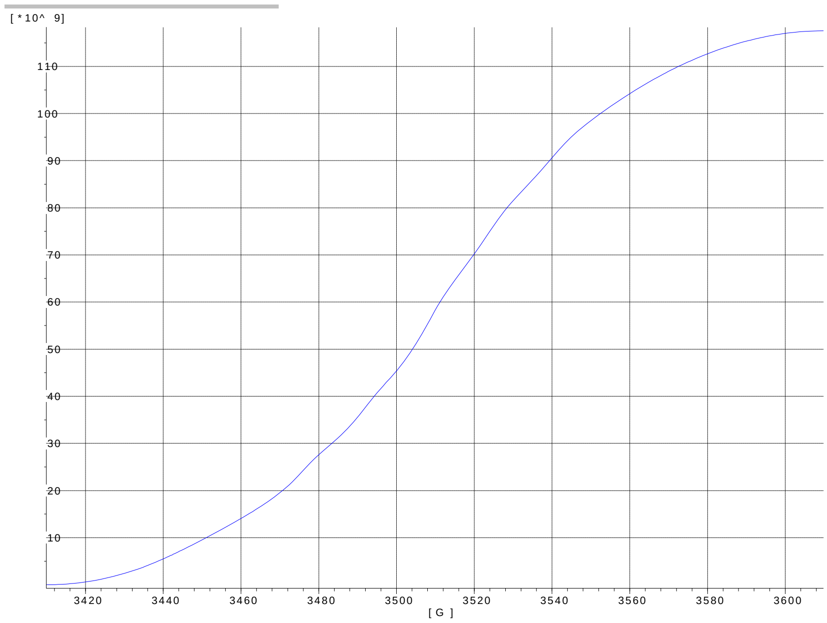

Supplementary Figure 17. Double integrate of Rh-diazo radical signal.

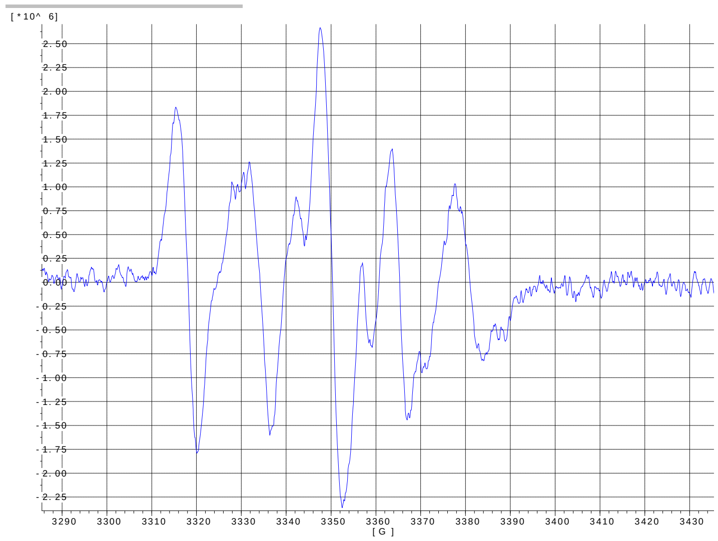

Supplementary Figure 18. EPR spectrum of Rh-diazo radical with Center Field 3360.56 G.

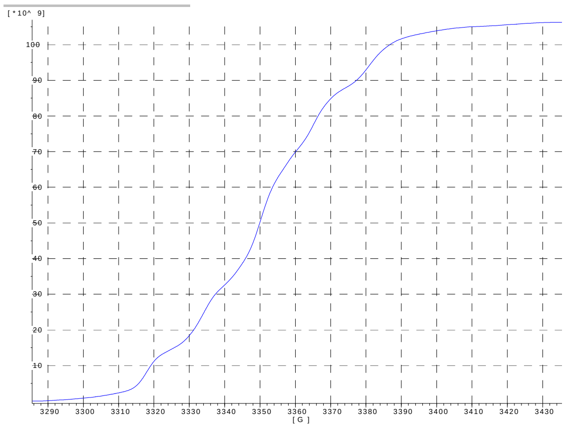

Supplementary Figure 19. Double integrate of Rh-diazo radical with Center Field 3360.56 G.

Analysis of the different EPR signals detected with Rh-diazo radical and Co-diazo radical in the presence of DMPO:

The 'quintet' EPR signals of Rh-diazo radicals come from the thermodynamical stabilities of Rh-diazomethane complexes and the stabilization effect of DMPO. For example, by the DFT-calculated results, N-centered Rh-diazo radical is a predominant structure and can be formed from diazocompounds and $[Rh^{I}Cl(cod)]_2$, but to the Co-diazo radical the predominant structure is metal-centered radical. Obviously, it is impossible to detect the quintet EPR spectra of Co-diazo radical.

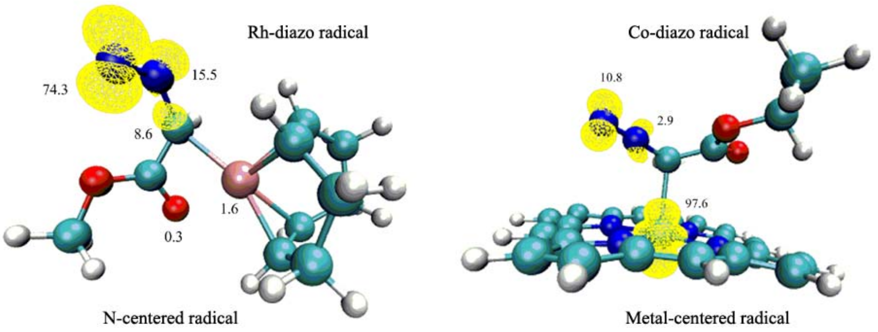

Supplementary Figure 20a. Spin density distributions of Rh- and Co-diazo radicals

However, the potential barrier between Rh-diazo radical and its TS is only 3.18 KJ/mol, which is quite easy to lose N_2 to transform into Rh-carbene radical. Experimentally, the product of $[Rh^{I}Cl(cod)]_2$-catalyzed C1 polymerization of diazoacetate is poly(carbethoxy carbene) (D. Hetterscheid, et al., ***J. Am. Chem. Soc.,*** 2006, 128, 9746-9752) but that of Pd-catalyzed polymerization of α-carbonyl diazomethane contains –N=N– subunit in its backbone (E. Ihara, et al., ***Macromolecules,*** 2005**,** *38,* 2101-2108), which indicates that Pd-diazo radical can act as either the precursors to Pd-carbene radical or the reactive monomer to take part in the polymerizations, but Rh-diazo radical mainly transforms into Rh-carbene radical that undergoes C1 polymerization to produce polycarbene. Clearly, Rh-diazo radical is not stable enough to be detected.

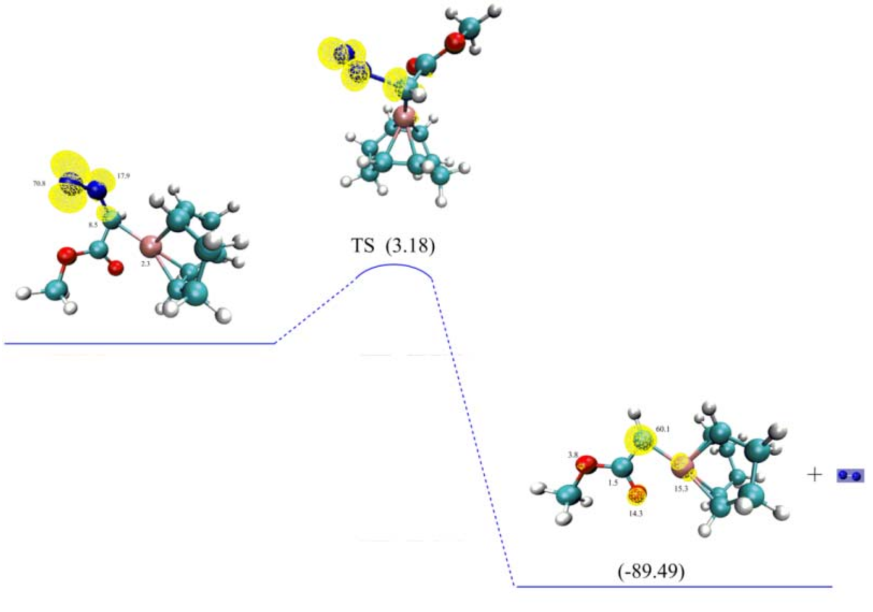

Supplementary Figure 20b. Potential barrier between Rh-diazo radical and its TS

In the presence of DMPO the linkage between Rh and carbon atom is relaxed by the interaction between Rh and negative oxygen ion of DMPO (c.f. E. Jellema, et al., ***J. Am. Chem. Soc.,*** 2007, 129, 11631-11641) and the linkage between the carbon atom of diazomethane and diazo group is reinforced, by which the nitrogen release is retarded. Obviously, Rh-diazo radical is stabilized by DMPO (DFT calculations indicate that the binding energy of DMPO-Rh-diazo radical complex (-139.82 KJ mol^{-1}) is 35.62 KJ mol^{-1} lower than that of Rh-diazo radical (-104.2 KJ mol^{-1})). The possible structure of DMPO-involved Rh-diazo radical is suggested as below:

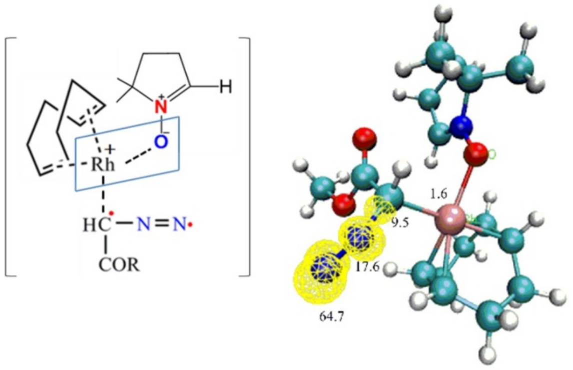

Supplementary Figure 20c. Possible structure of DMPO-Rh-diazo radical complex.

The steric effect of involved DMPO molecule prevents diazo radical being trapped by other DMPO molecules. So, Rh-diazo radical can be detected in the presence of DMPO but not captured by DMPO.

In the case of Co-diazo complexes, the spin density can transform from the metal atom into the –N=N–C– group to form a N-centered [M]-diazo radical, which is then captured by DMPO. The process is dynamically controlled and competes with the N_2-losing.

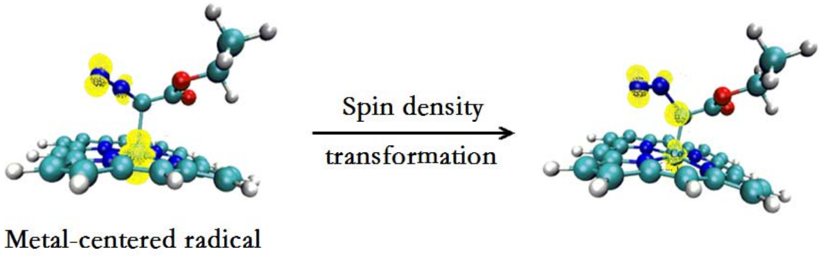

Supplementary Figure 21. Spin density transformation from the metal atom into –N=N–C– group.
